# Supplementary material for: Learning curve and surgical outcome of robotic assisted colorectal surgery with ERAS program
Source: Sci Rep. 2022 Nov 29;12:20566. doi: 10.1038/s41598-022-24665-w (PMC9709162; doi:10.1038/s41598-022-24665-w)
Supplement: Supplementary file 2 — Supplementary Information 2. [file 41598_2022_24665_MOESM2_ESM.zip › RAL Raw data-Ñ[▒K/136/OP N_ñw╝╨░O▒Kñσ.pdf]

W102 / W102

臺中榮民總醫院

手術室護理記錄

[redacted]

基本資料

- 室別： 開刀房-17室，手術科別： CRS，手術類別： 常規，日期： 2021/12/29
- 進入時間： 1525 ； 麻醉開始時間： 1530 ； Time Out時間： 1625 ； 劃刀時間： 1626 ； 手術結束時間： 2325 送出時間： 2355 ；
- 麻醉方式： GE

  診斷：rectum ca s/p SCRT+ FOLFOX

  手術名稱： 1.Lymphadenectomy, pelvic
2.Ileostomy
3.Robot Assisted Low Anterior Resection

  手術局部用藥：2%Xylocaine jelly

  術前預防性抗生素：
  - 抗生素名稱一： Veterin 1GM IVD由麻醉護理師給予
  - 時間： 1605
  - 上次簽章: 鄭鈞躍

  ，追加預防性抗生素：
  - 抗生素名稱一： Veterin 1GM IVD由麻醉護理師給予
  - 時間： 2005
  - 上次簽章: 鄭鈞躍
- 手術醫師： 林俊余，林陳彥，陳璿夫，蔡濬樟，張育豪
- 麻醉醫師： VS 洪至仁
- 刷手護士A ： 紀致均，時間： 1525，刷手護士B ： 王明盈，時間： 1525，接替刷手一： 林湘芸，時間： 1900，接替刷手二： 黃奎傑，時間： 2300

  流動護士A ： 鄭鈞躍，時間： 1525，接替流動一： 王瑜鳳，時間： 2000，接替流動二： 宋岱蓉，時間： 2100

皮膚狀況

A-V Shunt： 無

術前： 完整

記錄者： 上次簽章: 鄭鈞躍

術後： 不完整

部位(術後)：

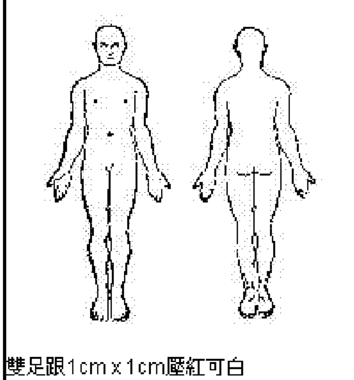

雙足跟1cm x 1cm壓紅可白

記錄者： 上次簽章: 宋岱蓉

手術準備

手術臥式 單一手術臥式

-
  - 手術臥式： 截石臥式
  - 使用附件： 溫毯 38 ℃，腳架
  - 皮膚準備： 10%Aq Betadine，Hibiscrub，2% Chlorhexidine
  - 電燒： 使用電燒: 電極片位置： 其它： 迴路電極墊，其它： nil，雙極電燒，記錄者： 上次簽章: 鄭鈞躍

止血帶T： 無

植入物

- Davinci Endo Stapler 45 Green x3/Colon
  ETHICON 29mm EEA/吻合處

, 記錄者: 上次簽章: 鄭鈞躍

標本送檢

- 細菌培養 無
- 細胞學 無
- 冰凍切片 無
- 病理標本
  組織名稱一： Rectum
  ,
  組織名稱二： IMA root
  ,
  組織名稱三： distal cut end
  , 更多組織名稱
  - 組織名稱四： left PLN
- 截肢 無

標本交付 標本： 4 袋 ， 標本： 0 盒 ， 標本： 0 瓶 ， 細胞學： 0 管 ， Culture Tube： 0 管 ， 被交付醫師： R 張育豪，

- 記錄者： 上次簽章: 宋岱蓉
- 時間： 2330

傷口術後敷料

傷口敷料：　　　　傷口敷料： 無

- 請於圖上標示部位：

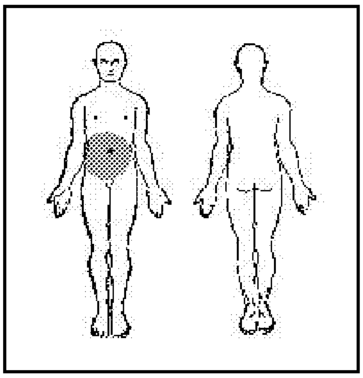

- 傷口敷料： Trocar洞:menpore

導管

導管	天數	導管資訊	插入日期	插入時間	更換日期	拔除日期	拔除時間	拔除原因	預計拔除/更換	預計拔除/更換日期
Chemo Port(1)	154	部位:鎖骨下; 象限:LU;	2021/07/30	1000						
Sump NG Tube(1)	2	號碼:16 Fr.; 深度:60cm; 部位:胃;	2021/12/29	1645						
Hemo Vac(1)	2	號碼:15 Fr.; 部位:骨盆腔;	2021/12/29	2230						
Foley(1)	2	號碼:16 Fr.; 部位:膀胱;	2021/12/29	1545						

用物計數

- 手術中出血量： 100 c.c.
- 手術中尿量： 350 c.c.
-
  - 其它： 無

用物計數： 多切口同時縫合

- 1.器械： 正確，2.縫針： 正確，3.刀片： 正確，4.紗布： 正確，5.腹部墊： 未使用，6.紗球： 未使用，7.PEANUTS ： 未使用，8.紗條： 未使用，9.絲帶： 未使用，10.針頭： 未使用，11.Vessel loop： 正確，12.Boots： 未使用，13.Jelco： 未使用，14.棉條： 未使用，
  - 第一次：
    - 刷手護士 ： 林湘芸
    - 流動護士： 上次簽章: 宋岱蓉
    - 時間： 2230

  ,
  - 第二次：
    - 刷手護士 ： 林湘芸
    - 流動護士： 上次簽章: 宋岱蓉
    - 時間： 2250

  ,
  - 第三次：
    - 刷手護士 ： 林湘芸
    - 流動護士： 上次簽章: 宋岱蓉
    - 時間： 2300

備註

備註： davinci 30度 XI scopy:No.1
davinci 0度 XI scopy:No.2

- 記錄者： 上次簽章: 鄭鈞躍
- 時間： 1530

填寫護理人員 宋岱蓉

正式摘要

W102 / W102

doctorRank:B
